# Catalytically defective receptor protein tyrosine kinase PTK7 enhances invasive phenotype by inducing MMP-9 through activation of AP-1 and NF-κB in esophageal squamous cell carcinoma cells

**DOI:** 10.18632/oncotarget.12303

**Published:** 2016-09-28

**Authors:** Won-Sik Shin, Yuri Hong, Hae Won Lee, Seung-Taek Lee

**Affiliations:** ^1^ Department of Biochemistry, College of Life Science and Biotechnology, Yonsei University, Seoul, Republic of Korea; ^2^ Department of Thoracic Surgery, Korea Cancer Center Hospital, Korea Institute of Radiological and Medical Sciences, Seoul, Republic of Korea

**Keywords:** PTK7, MMP-9, NF-κB, AP-1, esophageal squamous cell carcinoma (ESCC)

## Abstract

Protein tyrosine kinase 7 (PTK7), a member of the catalytically defective receptor protein tyrosine kinase family, is upregulated in various cancers including esophageal squamous cell carcinoma (ESCC). Here, we have explored the molecular mechanism of PTK7-dependent invasiveness in ESCC cells. PTK7 knockdown reduced gelatin degradation and MMP-9 secretion in cultures of ESCC TE-10 cells, and showed reduced levels of *MMP9* mRNA using real-time RT-PCR and luciferase reporter assays. PTK7 knockdown decreased not only phosphorylation of NF-κB, IκB, ERK, and JNK, but also nuclear localization of NF-κB and AP-1 consisting of c-Fos and c-Jun. Activation of AP-1 and NF-κB requires PTK7-mediated activation of tyrosine kinases, including Src. In addition, NF-κB activation by PTK7 involves the PI3K/Akt signaling pathway. PTK7-mediated upregulation of *MMP9* was also observed in other ESCC cell lines and in three-dimensional cultures of TE-10 cells. Moreover, MMP-9 expression positively correlated with PTK7 expression in ESCC tumor tissue. These findings demonstrate that PTK7 upregulates *MMP9* through activation of AP-1 and NF-κB and, thus increases invasive properties of ESCC cells.

## INTRODUCTION

Protein tyrosine kinase 7 (PTK7) consists of an extracellular domain with seven immunoglobulin-like loops, a transmembrane domain, and a tyrosine kinase domain that lacks detectable kinase activity; therefore, PTK7 is a member of the family of catalytically defective receptor protein tyrosine kinases (RPTKs) [1–4]. Homozygosity for a truncated PTK7 gene was perinatally lethal in mice and associated with severe developmental defects, including defective neural tube closure [5]. PTK7 mutant mice phenotypically overlap with known planar cell polarity (PCP) mutant mice and frogs, and PTK7 is genetically linked to Vangl2, a core PCP gene. PTK7 also interacts with canonical Wnt pathway proteins, including β-catenin, and triggers their target genes in *Xenopus* development, such as formation of Spemann's organizer [6]. Moreover, PTK7 interacts with Wnt5A, non-canonical Wnt/PCP ligand, and induces JNK activation during morphogenetic movements in *Xenopus* [7]. These findings suggest that PTK7 regulates PCP, canonical and non-canonical Wnt signaling pathways during development.

PTK7 is upregulated in esophageal squamous cell carcinoma (ESCC) [8], colorectal cancer [9, 10], and other cancers [11–15]. PTK7 enhances proliferation, survival, and migration of various cancer cells [8, 11, 13, 16]. PTK7 increases activation of ERKs, JNK, and p38 in ESCC and vascular endothelial cells [8, 17], and decreases expression of BAX and cleavage of caspase-3, −8, and −9 in cholangiocarcinoma [15]. In colon cancer and ovarian cancer, PTK7 sensitizes canonical Wnt and non-canonical Wnt/PCP pathways, respectively [6, 18]. However, PTK7 also has a tumor-suppressive role in some cancer types [19–22]. The mechanism(s) underlying the contradictory roles played by PTK7 in different cancer types is unclear. Recently, we demonstrated that PTK7 displays phenotypes ranging from oncogenic to tumor-suppressive depending on its concentration relative to those of its binding partners, such as kinase insert domain receptor (KDR) [17]. Our finding of a biphasic function of PTK7 explains in part the discrepancy in the expression-level-dependent oncogenic functions of PTK7.

In a previous report, we described increased PTK7 expression in tumor tissue of ESCC patients and its correlation with poor prognosis [8]. Moreover, PTK7 knockdown inhibited invasiveness and other oncogenic phenotypes of ESCC cells. In an attempt to identify a proteolytic enzyme responsible for the PTK7-mediated invasiveness, we performed fluorescent gelatin degradation assay and gelatin zymography. We identified matrix metalloproteinase (MMP)-9 as an enzyme responsible for the invasiveness, analyzed signaling pathways involved in induction of MMP-9, and described the molecular mechanism underlying PTK7-mediated invasiveness in ESCC TE-10 cells. We also demonstrate the correlation of PTK7 expression and MMP-9 induction in multiple ESCC cell lines and patients.

## RESULTS

### PTK7 knockdown inhibits gelatin degradation by reducing MMP-9 secretion in ESCC TE-10 cells

**Figure 1:**
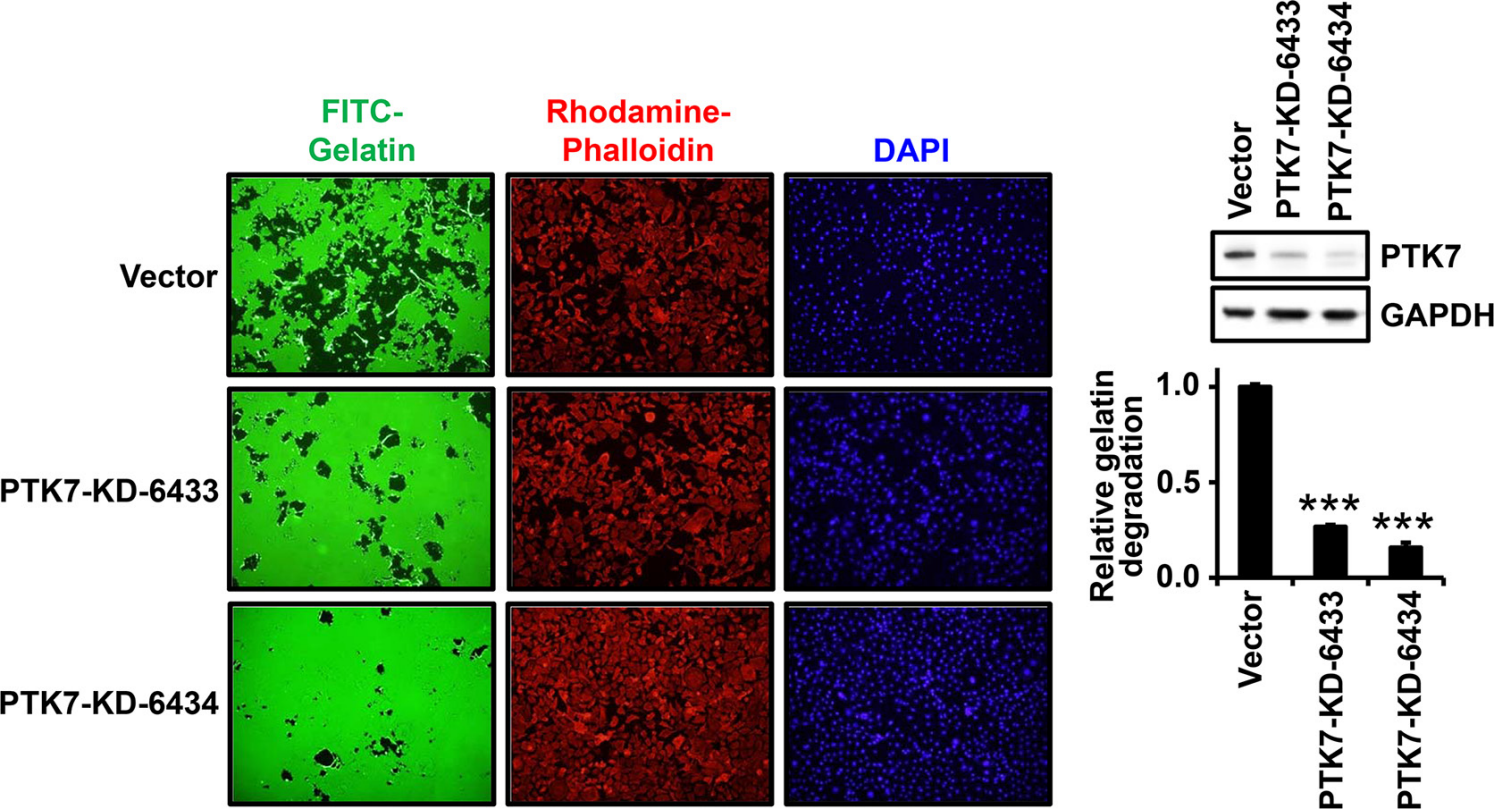
Effect of PTK7 knockdown on gelatin degradation by TE-10 cells Control vector-transfected and PTK7 knockdown (PTK7-KD-6433 and −6334) TE-10 cells were plated at 4 × 10^4^ cells/well of 24-well plate on FITC–gelatin-coated cover glasses and incubated for 48 h at 37°C. The cells were stained with rhodamine-phalloidin and DAPI, and analyzed by fluorescence microscopy (×100). Western blot on right shows PTK7 levels in control and PTK7 knockdown cells. GAPDH served as loading control. Relative gelatin degradation was shown as FITC-gelatin degraded area normalized to DAPI intensity of the sample referred to that of the control vector-transfected cells. ***P < 0.001 vs. control vector-transfected cells.

**Figure 2:**
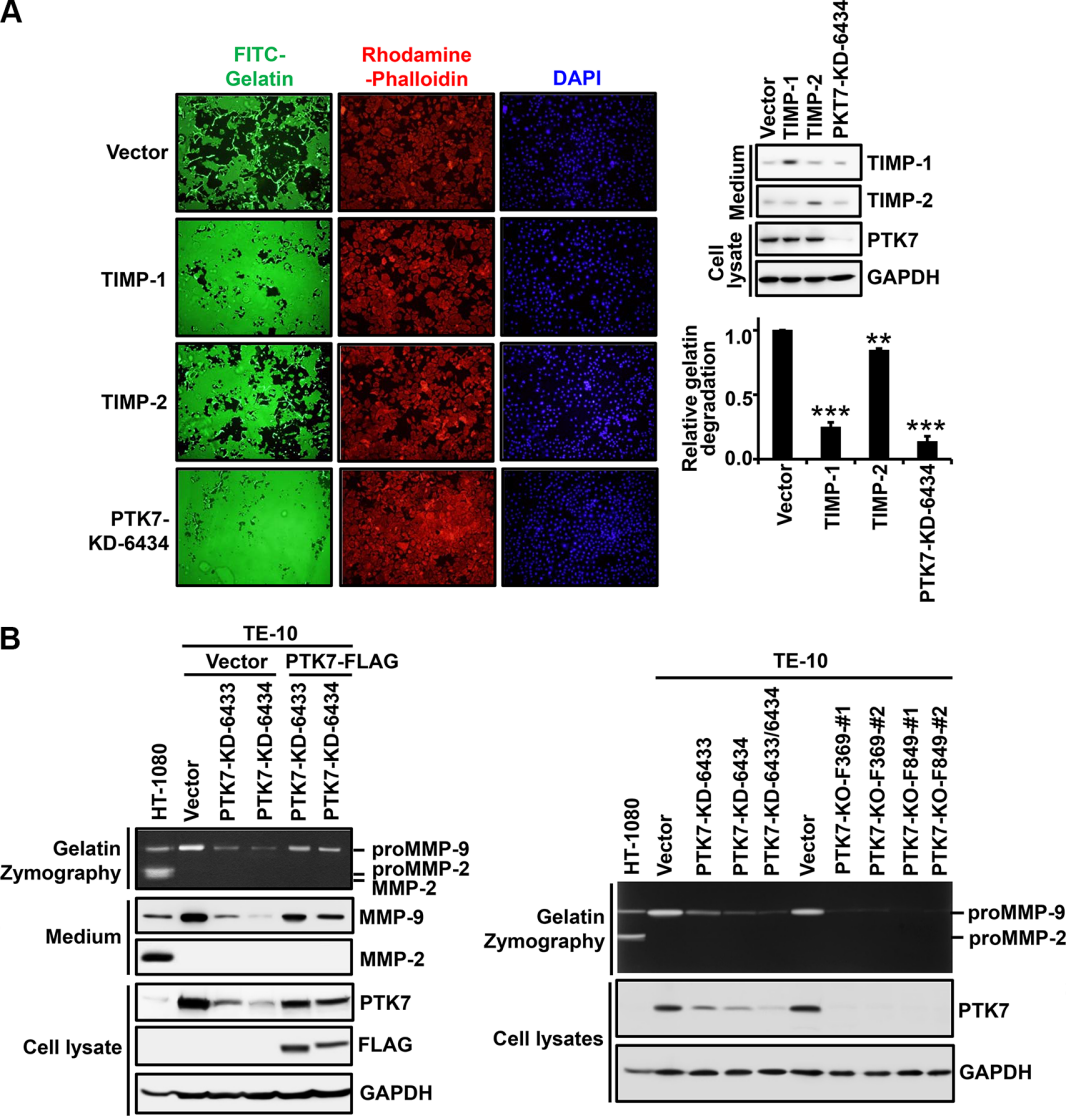
Identification of a gelatinase induced by PTK7 in TE-10 cells (**A**) TE-10 cells overexpressing TIMP-1 or TIMP-2 were grown on FITC–gelatin-coated coverslips, stained with rhodamine-phalloidin and DAPI, and analyzed by fluorescence microscopy (×100). Western blot on right shows TIMP-1 and TIMP-2 levels in conditioned medium and PTK7 level in cell lysates. Relative gelatin degradation was shown as FITC-gelatin degraded area normalized to DAPI intensity of the sample referred to that of the control vector-transfected cells. **P < 0.01, ***P < 0.001 vs. control vector-transfected cells. (**B**) Levels of secreted MMP-2 and MMP-9 and PTK7 were analyzed by gelatin zymography and western blotting in conditioned medium and cell lysates. PTK7 knockdown (PTK7-KD-6433 and 6434) TE-10 cells transfected with empty vector (Vector) or PTK7 overexpression vector (PTK7-FLAG) (left panel) and PTK7 knockdown (PTK7-KD-6433, 6434, and 6433/6434) or PTK7 knockout (2 cell lines of PTK7-KO-F369 and F849) TE-10 cells (right panel) were used. HT-1080 cell conditioned medium was used to show positions of MMP-9 and MMP-2.

We analyzed whether PTK7 stimulates focal proteolytic degradation of extracellular matrix (ECM) components in ESCC TE-10 cell cultures using a fluorescent gelatin degradation assay. Two lines of PTK7 knockdown cells, PTK7-KD-6433 and PTK7-KD-6434, showed significantly decreased degradation of FITC-labeled gelatin compared to control vector-transfected cells (Figure 1). To examine whether the gelatinases MMP-2 and MMP-9 are involved in PTK7-mediated gelatin degradation, extent of gelatin degradation was analyzed in TE-10 cells overexpressing tissue inhibitor of metalloproteases (TIMP)-1 and TIMP-2 (Figure 2A). TIMP-1 expression significantly reduced gelatin degradation to the similar extent as PTK7 knockdown in TE-10 cells. However, TIMP-2 expression inhibited gelatin degradation poorly in TE-10 cells. It is known that TIMP-1 inhibits both MMP-2 and MMP-9 and that TIMP-2 inhibits MMP-2, but not MMP-9 [23]. Thus, this observation suggests that PTK7-induced gelatin degradation is mediated by increased MMP-9 secretion in TE-10 cells.

To further confirm this finding, conditioned medium of control vector-transfected and PTK7-knockdown cells was analyzed by gelatin zymography and western blotting (Figure 2B). MMP-9 secretion was significantly decreased in PTK7 knockdown cells compared to control cells. Expression of exogenous PTK7-FLAG in the PTK7 knockdown cells restored MMP-9 secretion (Figure 2B, left panel). Moreover, gradual decrease of PTK7 expression using single and double transfection of two knockdown vectors is in parallel with decrease of MMP-9 secretion in TE-10 cells. PTK7 knockout almost completely abolished MMP-9 secretion (Figure 2B, right panel). MMP-2 secretion was not detected in ESCC TE-10 cells, regardless of PTK7 expression status. These results demonstrate that PTK7 is indispensable for MMP-9 expression in TE-10 cells.

### PTK7 shedding is not involved in PTK7-induced MMP-9 secretion in TE-10 cells

We previously reported that a cytosolic domain (CTF2) of PTK7 generated by sequential cleavage by ADAM17 and γ-secretase translocates into nucleus and enhances oncogenic phenotype of colon cancer cells [24]. However, the extracellular domain of PTK7, which can be produced by its shedding, was not detected in the conditioned medium of TE-10 cells (Supplementary Figure S1A). Incubation with GW280264X (ADAM 17/10 inhibitor) and/or DAPT (gamma-secretase inhibitor) to inhibit generation of PTK7-CTF2, did not change the secreted MMP-9 level in TE-10 cells (Supplementary Figure S1A). Therefore, we assume that PTK7-CTF2 is not produced in TE-10 cells. In addition, ectopic expression of PTK7-CTF2 did not change the secreted MMP-9 level although some of the expressed PTK7-CTF2 was detected in nucleus (Supplementary Figure S1B). Therefore, we conclude that PTK7-CTF2 does not affect induction of MMP-9 expression in TE-10 cells.

### PTK7 knockdown decreases transcription of *MMP9* mRNA in TE-10 cells

**Figure 3:**
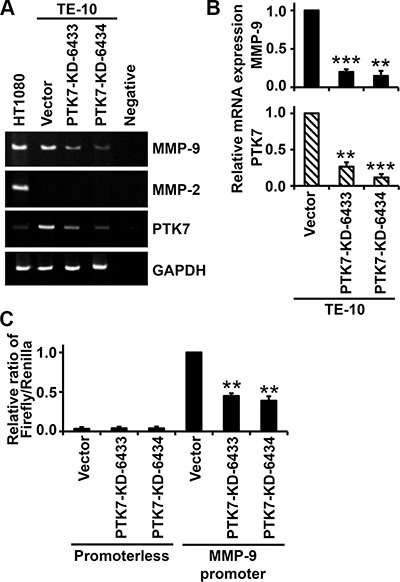
Effect of PTK7 knockdown on *MMP9* transcription in TE-10 cells (**A** and **B**) *MMP9* mRNA levels in control vector-transfected and PTK7 knockdown (PTK7-KD-6433 and −6434) TE-10 cells were analyzed by conventional RT-PCR (A) and real-time RT-PCR (B). (A) PCR products were separated by PAGE in 5% gels and visualized by ethidium bromide staining. (B) Relative expression levels of *MMP9* and *PTK7* mRNA determined by real-time PCR. **P < 0.01, ***P < 0.001 *vs.* those in control vector-transfected cells. (**C**) Control vector-transfected and PTK7 knockdown TE-10 cells were cotransfected with pRL-TK and pGL3-M9P-wt (*MMP9* promoter) or pGL3-Basic (promoterless). Luciferase activity is shown as ratio of firefly/*Renilla* luciferase activity. **P < 0.01 *vs.* luciferase activity in control vector-transfected TE-10 cells transfected with pGL3-M9P-wt.

To examine whether decreased MMP-9 secretion level is the result of decreased *MMP9* mRNA level, control vector-transfected cells and PTK7-knockdown cells were analyzed by reverse transcription (RT)-PCR and reporter assays. In PTK7-KD-6433 and PTK7-KD-6434 cells, *MMP9* mRNA levels were reduced to 20% and 15% and *PTK7* mRNA levels were reduced to 26% and 12%, respectively, compared to control cells (Figure 3B). Consistent with the absence of secreted MMP-2 (Figure 2B), *MMP2* mRNA was not detected in TE-10 cells (Figure 3A). Luciferase activity driven by the *MMP9* promoter was decreased to 45% and 39% of control values in PTK7-KD-6433 and 6434 cells, respectively (Figure 3C). These findings show that PTK7 upregulates *MMP9* at the transcriptional level.

### PTK7 knockdown inhibits phosphorylation of IκB, NF-κB, and mitogen-activated protein kinases (MAPKs) in TE-10 cells

**Figure 4:**
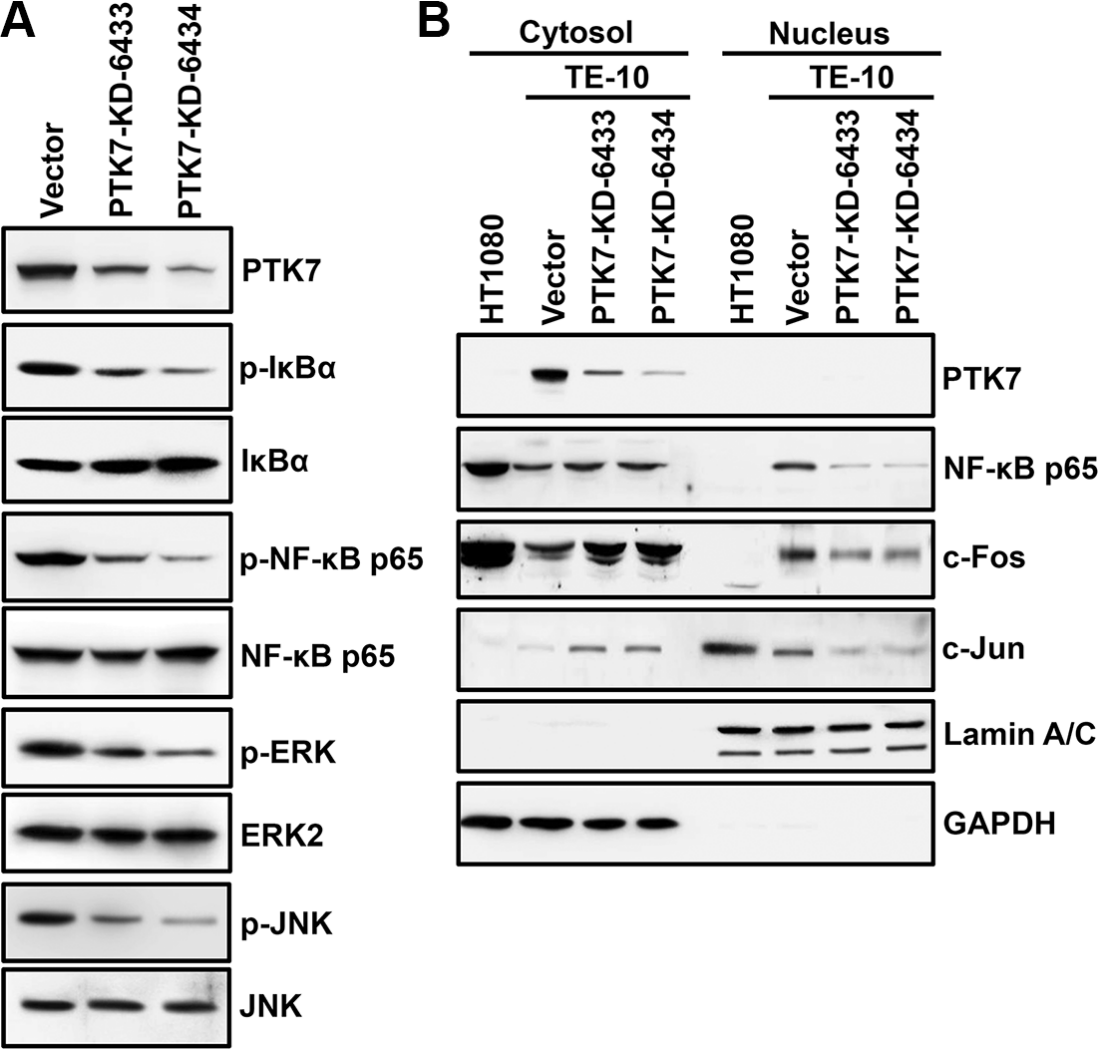
Effect of PTK7 knockdown on NF-κB and AP-1 activation in TE-10 cells Activation of NF-κB and AP-1 was analyzed in control vector-transfected and PTK7 knockdown (PTK7-KD-6433 and 6434) TE-10 cells. (**A**) Phosphorylation of NF-κB and IκB as indicators of NF-κB activation, and phosphorylation of ERK and JNK as indicators of activation of AP-1 were analyzed by western blotting. (**B**) Nuclear and cytosolic fractions were prepared from control and PTK7 knockdown cells. Levels of AP-1 complex components, c-Fos and c-Jun, and NF-κB were analyzed by western blotting. GAPDH and lamin A/C served as cytosolic and nuclear markers, respectively.

*MMP9* gene expression is increased primarily by transcription factors AP-1 and NF-κB [25]. Therefore, we analyzed phosphorylation of IκB and NF-κB, as indicators of NF-κB activation status, and of ERK and JNK, as indicators of AP-1 activation status, in PTK7 knockdown cells (Figure 4A). Phosphorylation of IκB, NF-κB, ERK, and JNK was reduced in the PTK7 knockdown cells, and nuclear levels of AP-1 consisting of c-Fos and c-Jun, and NF-κB were decreased in the PTK7 knockdown cells compared to control cells (Figure 4B). These observations show that PTK7 upregulates MMP-9 by activating the NF-κB and AP-1 signaling pathways.

### RPTKs and Src are involved in PTK7-mediated activation of NF-κB and AP-1 in TE-10 cells

**Figure 5:**
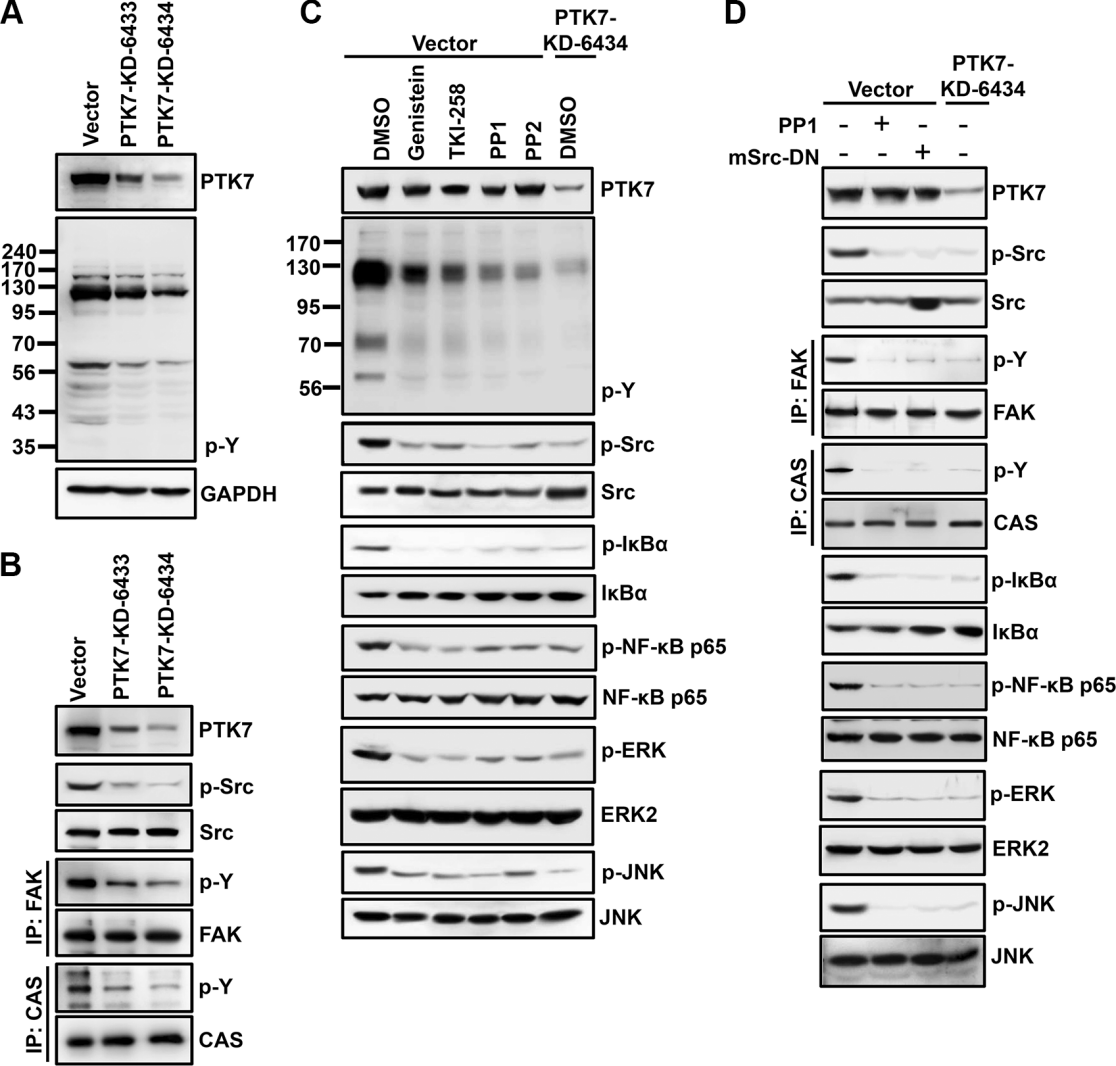
Involvement of PTK in PTK7-mediated activation of NF-κB and AP-1 in TE-10 cells (**A** and **B**) Tyrosine phosphorylation of cellular proteins (A) and p60^Src^, p125^FAK^, and p130^CAS^ (B) was analyzed by western blotting in control vector-transfected and PTK7 knockdown (PTK7-KD-6433 and 6434) TE-10 cells. Tyrosine phosphorylation of cellular proteins and p60^Src^ was detected by anti-phosphotyrosine (4G10) and anti–phospho-Src family (Tyr416) antibodies, respectively. Tyrosine phosphorylation of p125^FAK^ and p130^CAS^ was monitored by immunoprecipitation with anti-FAK and anti-CAS antibodies, respectively, and western blotting with anti-phosphotyrosine (4G10) antibody. (**C**) Subconfluent control vector-transfected and PTK7 knockdown (PTK7-KD-6434) TE-10 cells were incubated with genistein (100 μM, pan-PTK inhibitor), TKI-258 (200 nM, multi-targeted RTK inhibitor), PP1 or PP2 (10 μM, Src family kinase inhibitors), or DMSO (vehicle) for 30 min. (**D**) Control vector-transfected cells were transfected with dominant-negative mouse Src expression construct (mSrc-DN). Levels of total and phosphorylated forms of the indicated signaling molecules were analyzed by western blotting.

To elucidate the signal transduction pathway involved in PTK7-mediated NF-κB activation, phosphorylation of various signaling proteins in the presence of signaling inhibitors was analyzed in control and PTK7 knockdown cells. Total tyrosine phosphorylation was decreased in PTK7-KD-6433 and −6434 cells compared to control vector cells (Figure 5A). Because a dramatic decrease in tyrosine phosphorylation was detected in proteins of approximately 60 kDa and 125–130 kDa, phosphorylation levels of p60^Src^, p125^FAK^, and p130^CAS^ were analyzed. Phosphorylation of all three proteins was decreased in PTK7 knockdown cells (Figure 5B). Incubation of control vector-transfected TE-10 cells with a pan-PTK inhibitor (genistein), a multi-target RPTK inhibitor (TKI-258), and Src family kinase inhibitors (PP1 and PP2) decreased not only tyrosine phosphorylation of total cellular proteins and Src, but also phosphorylation of IκB, NF-κB, ERK, and JNK, to the same extent as seen in PTK7 knockdown cells treated with vehicle control dimethyl sulfoxide (DMSO) (Figure 5C). Moreover, expression of dominant-negative mouse Src (mSrc-DN) decreased phosphorylation of Src, IκB, NF-κB, ERK, and JNK, as much as the levels in PP1-treated or PTK7-knockdwon TE-10 cells (Figure 5D). These results suggest that PTK7 activates AP-1 and NF-κB signaling pathways through RPTKs and Src in TE-10 cells.

### PI3K and Akt are required for PTK7-mediated NF-κB activation in TE-10 cells

**Figure 6:**
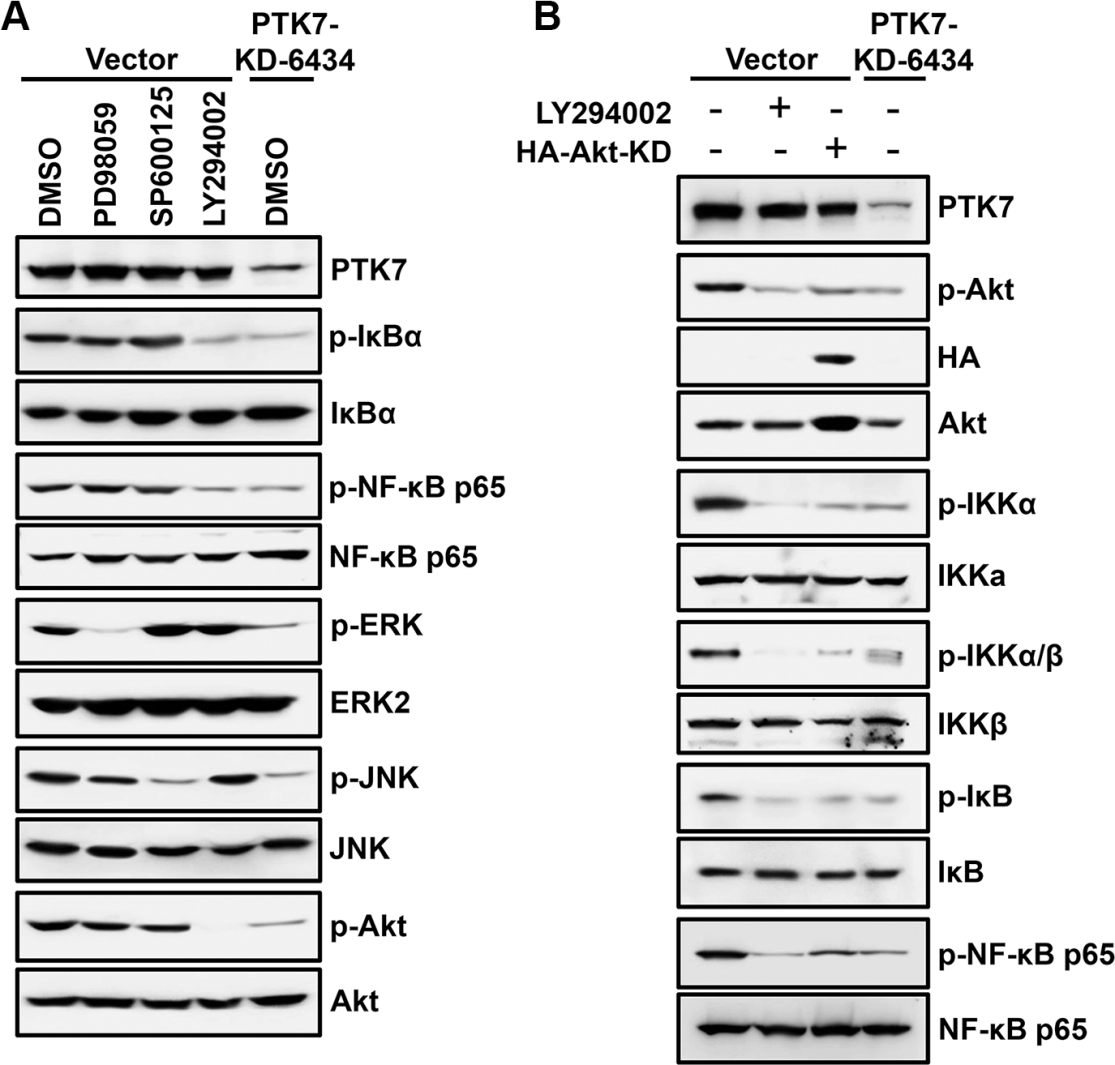
Effect of signaling blockade on the PTK7-mediated NF-κB activation in TE-10 cells (**A**) Subconfluent control vector-transfected and PTK7 knockdown (PTK7-KD-6434) TE-10 cells were incubated with PD98059 (30 μM, MEK inhibitor), SP600125 (10 μM, JNK inhibitor), LY294002 (10 μM, PI3K inhibitor), or DMSO (vehicle) for 30 min. Levels of total and phosphorylated forms of the indicated signaling molecules were analyzed by western blotting. (**B**) Control vector-transfected cells were transfected with dominant-negative Akt expression construct (HA-Akt-KD) or incubated in the presence or absence of LY294002 (10 μM) for 30 min. Levels of total and phosphorylated Akt, IKKα (Thr23), IKKα/β (Ser176/180), and IκB in the cells were compared with the ones in PTK7 knockdown (PTK7-KD-6434) cells by western blotting.

The PI3K/Akt pathway activates NF-κB through phosphorylation of the IκB kinase (IKK) complex in colorectal cancer cell [26]. In TE-10 cells, PTK7 knockdown decreased phosphorylation of Akt, IκB, and NF-κB (Figure 6A). As expected, PI3K inhibitor LY294002 decreased phosphorylation of IκB and NF-κB in control vector-transfected cells, whereas MEK inhibitor PD98059 and JNK inhibitor SP600125 did not inhibit phosphorylation of either protein. In PTK7 knockdown cells, phosphorylation of Akt, IKKα (Thr23), IKKα/β (Ser176/180), and IκB was significantly decreased, to the same extent as observed in LY294002-treated and dominant-negative Akt-overexpressing TE-10 cells (Figure 6B). These findings indicate that PTK7 enhances NF-κB phosphorylation through the PI3K-Akt signaling pathway in ESCC TE-10 cells.

### Blockade of PTK7-induced signaling pathways attenuates *MMP9* upregulation in 2D and 3D cultures of TE-10 cells

**Figure 7:**
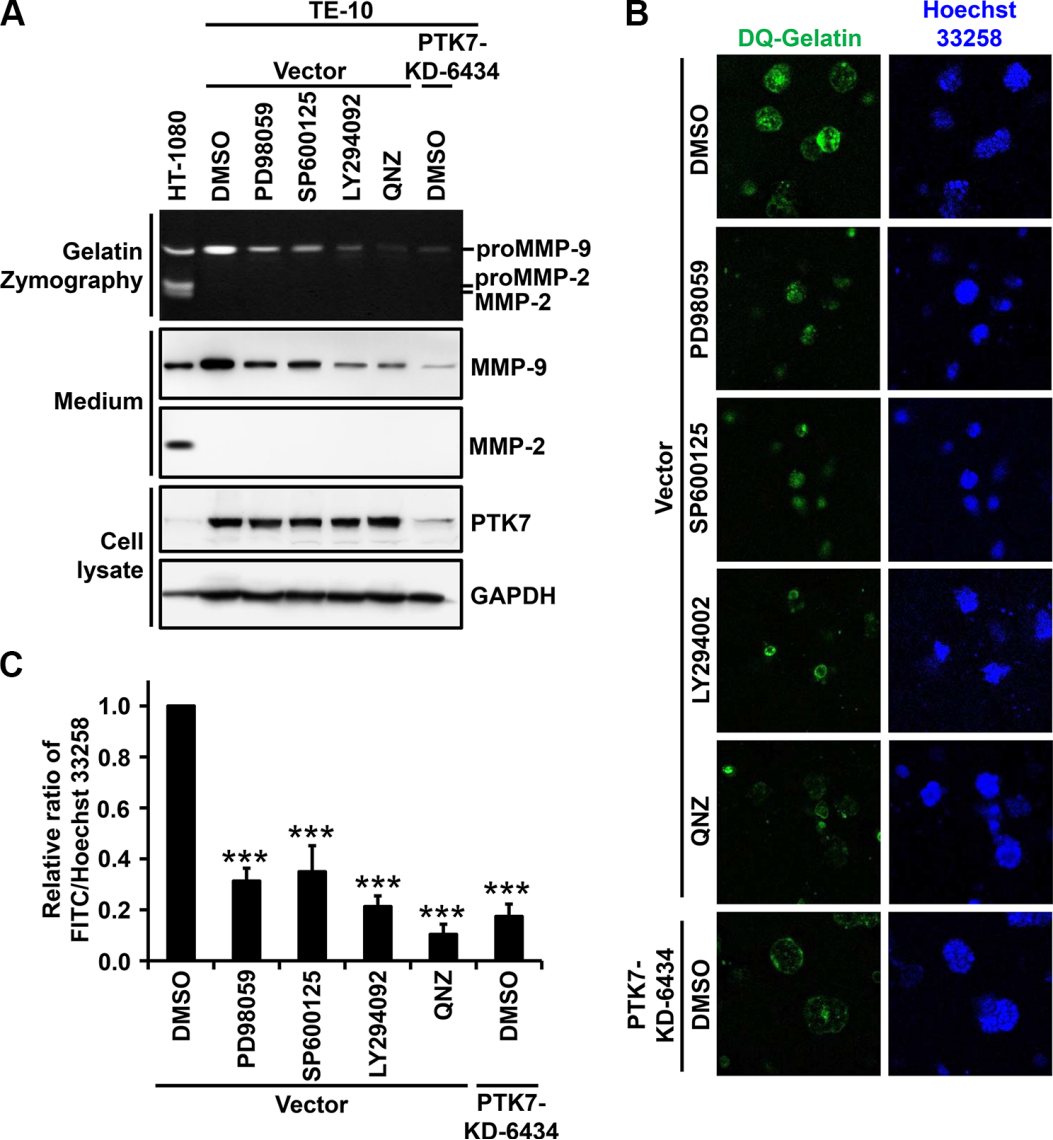
Effect of AP-1 and NF-κB signaling blockade on PTK7-mediated MMP-9 secretion in 2D and 3D TE-10 cell cultures (**A**) Subconfluent control vector-transfected and PTK7 knockdown (PTK7-KD-6434) TE-10 cells were incubated in serum-free medium containing PD98059 (30 μM), SP600125 (10 μM), LY294002 (10 μM), QNZ (10 μM, NF-κB inhibitor), or DMSO (vehicle) for 24 h. Levels of secreted MMP-9 and MMP-2 in the medium were analyzed by gelatin zymography and western blotting. Levels of PTK7 and GAPDH (loading control) in cell lysates were analyzed by western blotting. HT-1080 conditioned medium served to show positions of MMP-9 and MMP-2. (**B** and **C**) Control vector and PTK7 knockdown cells were cultured for 24 h within a 3D Matrigel matrix containing quenched fluorogenic DQ Gelatin in the presence of PD98059 (30 μM), SP600125 (10 μM), LY294002 (10 μM), QNZ (10 μM), or DMSO (vehicle). Nuclei were counterstained with Hoechst 33258, and cells were analyzed by confocal fluorescence microscopy (×200) (B) and gelatinolytic activity in the cultures was determined and expressed as the ratio of fluorescein (resulting from DQ-gelatin cleavage)/Hoechst 33258 fluorescence intensity (C). ***P < 0.001 vs. DMSO-treated control vector-transfected cells.

To confirm that PTK7-induced ERK, JNK, PI3K/Akt, and NF-κB signaling pathways are responsible for PTK7-mediated *MMP9* upregulation, MMP-9 expression and gelatin degradation were analyzed in 2D and 3D cultures of TE-10 cells in the presence of inhibitors of MEK, JNK, PI3K, and NF-κB. In 2D culture, treatment of cells with all tested inhibitors, as well as PTK7 knockdown, significantly decreased the level of secreted MMP-9 (Figure 7A). In 3D culture, intensity of fluorescence generated by degradation of fluorogenic DQ-gelatin was reduced by treatment with MEK, JNK, PI3K, and NF-κB inhibitors to 31%, 35%, 21%, and 10%, respectively, of control values (Figure 7B and 7C). In PTK7-KD-6434 cells, fluorescence intensity was reduced to 17% of control. These results demonstrate that PTK7-mediated transactivation of the *MMP9* gene occurs by AP-1 activation, which is associated with activation of ERK and JNK, and by NF-κB activation, which requires activation of PI3K.

### PTK7 expression is correlated with MMP-9 expression in other cells and tumor tissue of ESCC

**Figure 8:**
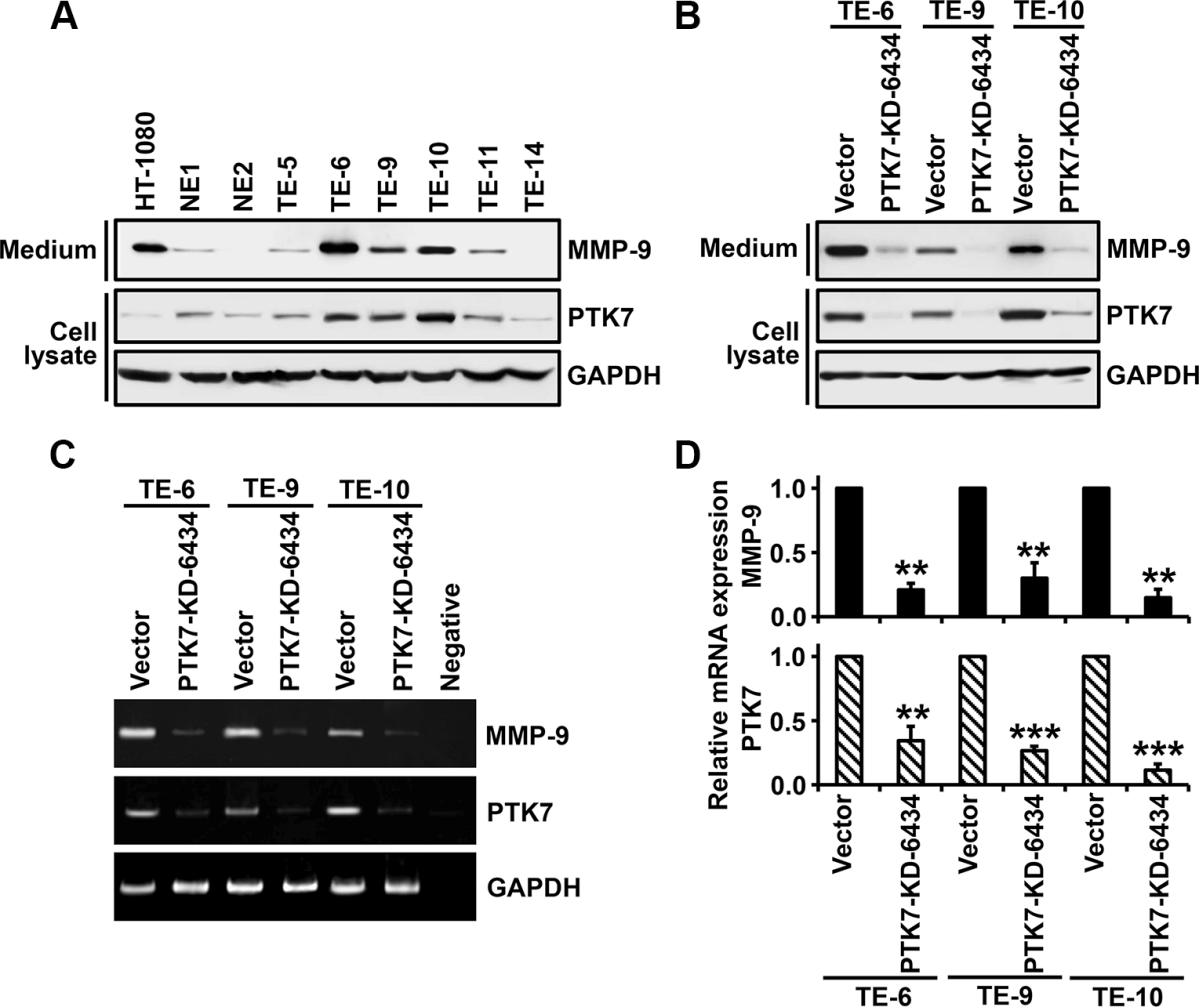
Analysis of PTK7-dependent MMP-9 expression in esophageal epithelial and ESCC cell lines In non-neoplastic esophageal epithelial cell lines (NE1 and NE2) and ESCC cell lines (TE-5, 6, 9, 10, 11, and 14) (**A**) and control vector-transfected and PTK7 knockdown (PTK7-KD-6434) TE-6, −9, and −10 cells (**B**), levels of secreted MMP-9 levels in the medium and levels of PTK7 and GAPDH in cell lysates were analyzed by western blotting. HT-1080 cell lysates served as an MMP-9–positive control. *MMP9* and *PTK7* mRNA levels were analyzed by conventional (**C**) and real-time (**D**) RT-PCR in control and PTK7 knockdown TE-6, −9, and −10 cells. **P < 0.01, ***P < 0.001 *vs.* level in control vector cells.

We analyzed levels of PTK7 and secreted MMP-9 in non-neoplastic esophageal epithelial cell lines and ESCC cell lines (Figure 8A). Both PTK7 and MMP-9 were expressed at a low level in the non-neoplastic esophageal epithelial cell lines NE1 and NE2. In the ESCC cell lines TE-5, TE-6, TE-9, TE-10, TE-11, and TE-14, levels of MMP-9 secretion varied. Interestingly, cells showing elevated PTK7 expression also showed elevated MMP-9 secretion (Figure 8A). PTK7 knockdown in TE-6, TE-9, and TE-10 cells reduced levels of secreted MMP-9 (Figure 8B). In PTK7 knockdown TE-6, TE-9, and TE-10 cells, PTK7 mRNA levels were decreased to 34%, 27%, and 12% respectively, of control values, and in these cells *MMP9* mRNA levels were decreased to 20%, 30%, and 14%, respectively, of control (Figure 8C and 8D). Thus, we are confident that PTK7 enhances *MMP9* expression at the transcriptional level in other ESCC cells as well as in TE-10 cells.

**Figure 9:**
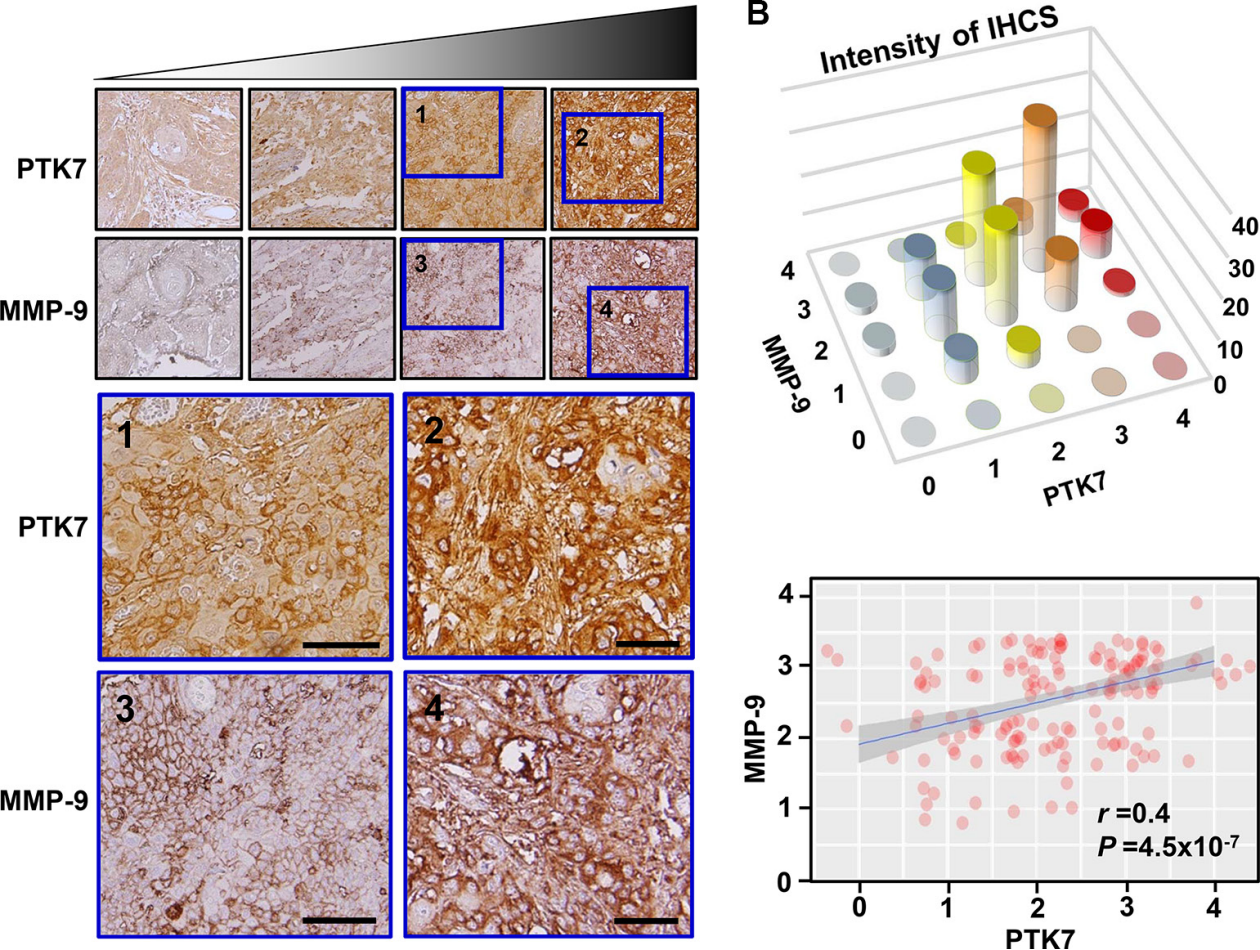
Relationship between PTK7 and MMP-9 expression based on immunohistochemical analysis of ESCC tissue (**A**) Representative images of immunohistochemical staining of PTK7 and MMP-9 in serial sections of each ESCC tissue block (×200). Areas inside blue boxes were shown at a higher resolution. Scale bar indicates 50 μm. (**B**) Distribution (top) and linear relationship determined by Spearman's rank correlation test (bottom) of PTK7 and MMP-9 staining intensity in 155 ESCC tissue samples. *r* and *P* values of Spearman's rank correlation are shown.

To investigate whether PTK7 expression was correlated with that of MMP-9 *in vivo*, tumor tissue from 155 ESCC patients was immunohistochemically stained with anti-PTK7 and anti-MMP-9 antibodies and staining intensity was analyzed. Representative images of PTK7 and MMP-9 staining in each tissue block are shown in Figure 9A. PTK7 and MMP-9 are enriched at pericellular region of cancer cells but not around normal cells. Spearman's rank correlation analysis showed that PTK7 expression was significantly correlated with MMP-9 expression (Figure 9B; Spearman's rank: *r* = 0.4 and *P* = 4.5 × 10^−7^). These findings support the idea that PTK7 expression is responsible for the induction of MMP-9 in ESCC tumor tissue.

## DISCUSSION

In this study, we found that PTK7 knockdown reduced focal degradation of underlying gelatin in TE-10 cell cultures. MMP-9 is the major enzyme responsible for PTK7-dependent gelatin degradation, and PTK7 induces *MMP9* at the transcriptional level. Transactivation of the *MMP9* gene is stimulated primarily by transcription factors AP-1 and NF-κB [27]. We found that PTK7 increased nuclear levels of c-Fos and c-Jun through activation of ERK and JNK and increased the level of activated NF-κB by phosphorylation and degradation of IκB and phosphorylation of RelA/p65.

It is interesting to understand how PTK7 activates MAPKs and IKK to activate AP-1 and NF-κB. Knockdown of PTK7 decreased tyrosine phosphorylation of cellular proteins in TE-10 cells. Although PTK7 is a catalytically defective RPTK, it might recruit protein tyrosine kinases and enhances downstream signaling pathways. It was well known that HER3, a catalytically defective RPTK, can heterodimerize with other EGFR family members upon ligand binding such as neuregulin and activates downstream signaling proteins such as Akt and Erk [28]. We have previously demonstrated that PTK7 binds to and activates KDR, one of VEGF receptor [17]. However, KDR expression was not detectable in TE-10 cells. Nevertheless, pan-PTK inhibitor genistein and multi-target RPTK inhibitor TKI-258 inhibited phosphorylation of ERK, JNK, IκB, and NF-κB. Thus, RPTKs other than KDR might work with PTK7 for downstream activation. We also found that PTK7 knockdown decreases phosphorylation of Src and Src substrate CAS [29]. Indeed, treatment of Src family kinase inhibitors PP1 and PP2 and expression of dominant-negative Src decreased activation of ERK, JNK, IκB, and NF-κB to the same extent as PTK7 knockdown. It was reported that PTK7 coprecipitates with Src and enhances Src activity [30]. In addition, Src is known to activate NF-κB, as well as ERKs and JNK [31, 32]. These reports support the idea that Src may be a PTK involved in PTK7-mediated activation of AP-1 and NF-κB.

**Figure 10:**
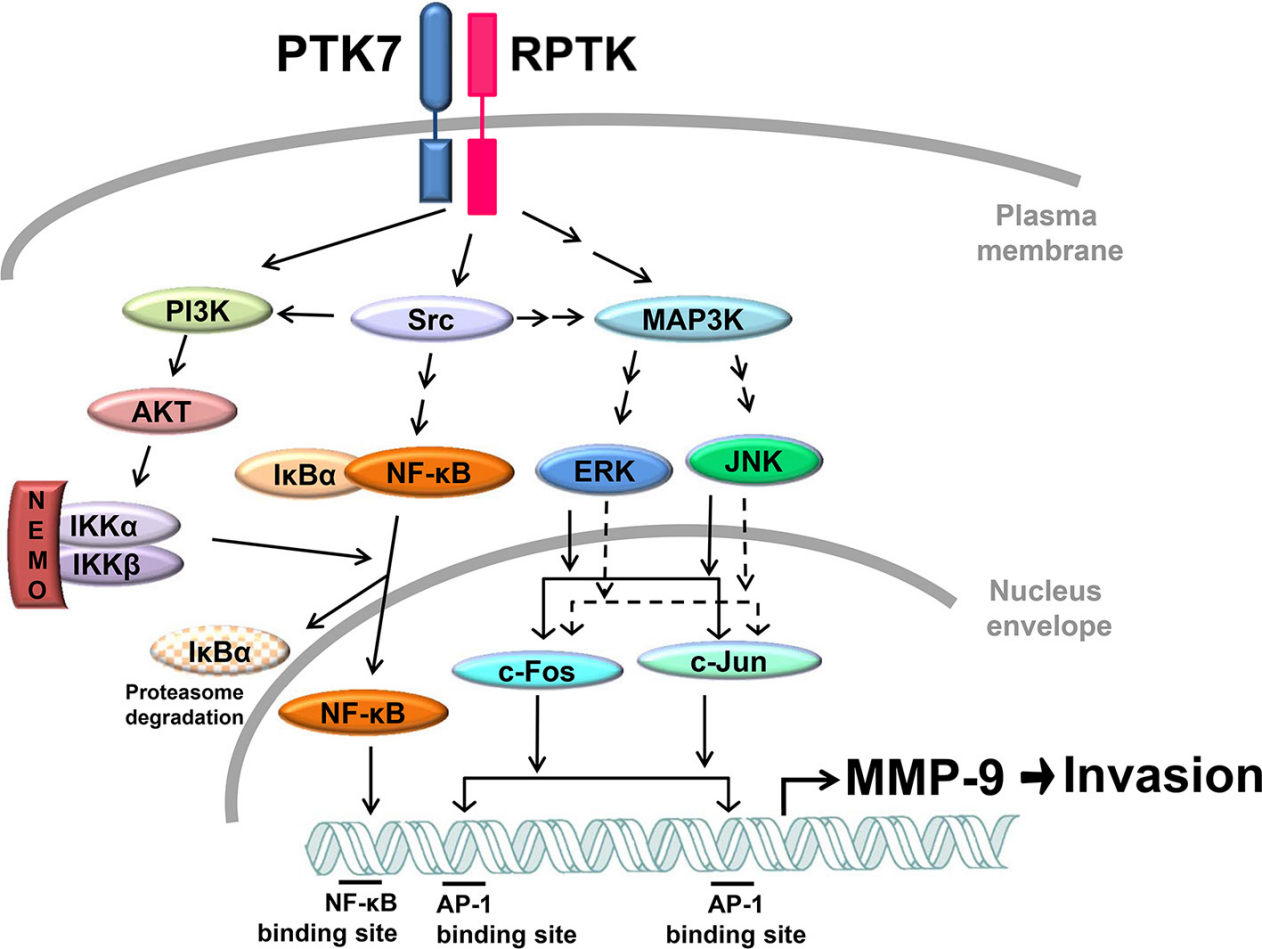
Proposed model for PTK7-mediated MMP-9 induction and invasiveness in TE-10 cells PTK7 activates the PI3K/AKT and Ras/MAPK pathways via activation of unidentified RPTK and Src. The PI3K–Akt–IKK signal cascade activates NF-κF. ERK and JNK activate AP-1 complex components c-Fos and c-Jun. NF-κB and AP-1 transactivate *MMP9* and enhance invasive phenotype of ESCC cells. Solid and dashed arrows indicate activation at the protein and transcriptional levels, respectively.

Interestingly, we found that PTK7-mediated phosphorylation of IκB and NF-κB was inhibited by a PI3K inhibitor, LY294002. IKKα is phosphorylated at Thr23 by Akt, and IKKα/β is phosphorylated at Ser176/180 by NIK and TAK1 or autophosphorylated by the IKK complex [33]. In our study, phosphorylation of IKKα at Thr23 and of IKKα/β at Ser176/180 was decreased by LY294002 treatment and overexpression of dominant-negative Akt. Thus, the PTK7-activated PI3K/Akt pathway appears to activate IKK in ESCC TE-10 cells. The signaling pathways involved in PTK7-induced MMP-9 expression are summarized in Figure 10.

We have shown that the pro-invasive effect of PTK7 occurs through enhanced MMP-9 secretion in 2D culture conditions. *In vivo*, cells are surrounded by ECM and grow or migrate through ECM proteolysis [34]. Use of a 3D culture system that incorporates Matrigel and DQ-gelatin to mimic *in vivo* conditions, we demonstrated that PTK7 knockdown, as well as inhibition of MEK, JNK, PI3K, and NF-κB activation, significantly inhibited degradation of ECM components.

Gelatinase activity is frequently increased in ESCC and is correlated with tumor invasion and metastasis [35]. We previously demonstrated that PTK7 expression is related to poor prognosis of ESCC patients [8]. Here, we showed that PTK7 expression is required for transcription and secretion of MMP-9 in multiple ESCC cell lines that express PTK7. Although *MMP9* induction is controlled by various stimuli and signaling pathways, we have shown that PTK7 expression is positively correlated with MMP-9 expression in samples of ESCC tumor tissue from 155 patients.

We showed that PTK7 enhances invasiveness through MMP-9 induction by AP-1 and NF-kB activation. Chemoresistant cancer cells often show NF-κB activation, and NF-κB knockdown strengthens the effect of chemotherapeutic agent 5-FU on cell death in cancer cells [36–38]. Consistently, we have shown that PTK7 knockdown sensitizes cells to 5-FU–induced death [8]. These data suggest the possibility that inhibition of PTK7 function reduces NF-κB activation and enhances the effect of 5-FU in chemoresistant cancer cells. Taken together, the data show that PTK7 plays a role in tumorigenesis, invasiveness, and chemoresistance. Thus, attenuation of PTK7 function would be a valuable therapeutic means to control ESCC and other cancers that express PTK7.

## MATERIALS AND METHODS

### Reagents and antibodies

Quenched fluorogenic DQ Gelatin and rhodamine-conjugated phalloidin were purchased from Thermo Fisher Scientific (Waltham, MA, USA). Fluorescein isothiocyanate (FITC)-conjugated gelatin, anti-FLAG antibody, and 4′,6-diamidino-2-phenylindole (DAPI) were purchased from Sigma-Aldrich (St. Louis, MO, USA). Genistein, PP1, PP2, and LY294002 were purchased from AG Scientific (San Diego, CA, USA). TKI-258 was purchased from ApexBio (Houston, TX, USA). PD98059 and SP600125 were purchased from Tocris Bioscience (Bristol, UK). QNZ and antibodies against phospho-ERK, ERK2, c-Fos, lamin A/C, phospho-IKKα (Thr23), IKKα, NF-κB p65, CAS, FAK, Src, and HA tag were purchased from Santa Cruz Biotechnology (Santa Cruz, CA, USA). Antibodies against phospho-JNK, JNK, c-Jun, phospho-Src family (Tyr416), phospho-Akt (Ser473), Akt, phospho-IKKα/β (Ser176/180), IKKβ, phospho-IκBα (Ser32), IκBα, phospho-NF-κB p65 (Ser536), and MMP-9 were purchased from Cell Signaling Technology (Danvers, MA, USA). Anti-phosphotyrosine antibody (clone 4G10) and anti–MMP-2 antibody were purchased from Millipore (Billerica, MA, USA). Anti-GAPDH antibody was purchased from AbClone (Seoul, Korea). Horseradish peroxidase-conjugated goat anti-mouse IgG, rabbit IgG, and rabbit anti-goat IgG were purchased from KOMA Biotech (Seoul, Korea). Anti-PTK7 antibody was described previously [39].

### Cell culture

Human ESCC TE-5, TE-6, TE-9, TE-10, TE-11, and TE-14 cells were obtained from the RIKEN BioResource Center (Tsukuba, Japan) and human fibrosarcoma HT-1080 cells was obtained from Korean Cell Line Bank (Seoul, Korea). These cells were maintained in Dulbecco's modified Eagle medium (DMEM; Gibco/Thermo Fisher Scientific) supplemented with 10% fetal bovine serum (FBS). Non-neoplastic esophageal epithelial cell lines NE1 [40] and NE2 [41] were provided by Professor G.S.W. Tsao (University of Hong Kong, Hong Kong SAR, China). These cells were maintained in a 1:1 mixture of Epilife medium (Gibco/Thermo Fisher Scientific) and Defined Keratinocyte-SFM (Gibco/Thermo Fisher Scientific) containing 100 U/ml penicillin and 100 μg/ml streptomycin at 37°C in a humidified atmosphere of 5% CO_2_ and 95% air.

### Expression vectors, knockdown vectors, and knockout vectors

Expression vectors pcDNA3.1-TIMP-1 [42] and pcDNA3.1-TIMP-2 [43] encoding human TIMP-1 and TIMP-2, respectively, and pcDNA3-hPTK7-FLAG [17] encoding human PTK7 with a C-terminal Flag tag were described previously. Expression vector encoding K297R/Y529F dominant-negative mouse Src (pLNCX-mSrc [K297R/Y529F]) was generated by site-directed mutagenesis using pLNCX-mSrc, which was a generous gift from Professor E.-S. Oh (Ewha Womans University, Korea), as a template and primer pairs listed in Supplementary Table S1. Expression vector encoding HA-tagged K179M kinase-deficient AKT (pCMV6-HA-Akt-KD [K179M]) was a kind gift of Professor K.-Y. Choi (Yonsei University, Korea). Constructs pLKO.1-shRNA-PTK7–6433 and −6434 for human PTK7 knockdown vectors and pLKO.1-control (Sigma-Aldrich) were described previously [8]. Constructs LentiCRISPRv2-sgRNA-PTK7-F369 and –F849 for human PTK7 knockout vectors were generated by cloning of oligonucleotide sequences listed in Supplementary Table S2 into LentiCRISPRv2 vector (Addgene, Cambridge, MA, USA).

### Generation of PTK7 knockdown and PTK7 knockout ESCC cells

Production of PTK7 knockdown and PTK7 knockout lentiviruses and infection into ESCC cells were performed as described previously [8]. Puromycin-resistant cells were enriched by incubation of the cultures with 2.5 μg/ml puromycin for 14 d. A mixed culture of PTK7 knockdown cells and individual clones of PTK7 knockout cells were maintained in the presence of 1 μg/ml puromycin.

### Transfection

Transfection of expression vectors into cells was carried out using Lipofectamine 2000 (Thermo Fisher Scientific) according to the manufacturer's instructions. Briefly, the media of subconfluent cells in 6-cm dish were replaced with 4 ml of Opti-MEM (Gibco/Thermo Fisher Scientific). A mixture of 4 μg of expression vector and 8 μl of Lipofectamine 2000 in 0.4 ml Opti-MEM was incubated for 30 min at room temperature and was added to the cells. After 6-h incubation, the media were changed to 10% FBS DMEM and the cells were analyzed for reporter assay at 24 h and for transient expression at 48 h after transfection.

### Fluorescent gelatin degradation assay

Cross-linked FITC–gelatin-coated coverslips were prepared as described previously [44]. Briefly, acid-washed glass coverslips were coated with 20 μg/ml FITC-gelatin for 2 h at 37°C. The coverslips were washed with phosphate-buffered saline (PBS), cross-linked with 0.5% glutaraldehyde for 15 min, and then quenched with 5 mg/ml sodium borohydride for 3 min at 25°C. The FITC-gelatin–coated coverslips were washed with PBS again and incubated with 5% FBS in DMEM for 1 h at 37°C. Cells (4 × 10^4^/well of 24-well plate) were seeded on the coverslips and incubated for 48 h at 37°C to allow gelatin degradation. The cells were fixed with 3.7% paraformaldehyde and treated with 1% Triton X-100. Actin filaments were stained with rhodamine-phalloidin (500 ng/ml). Nuclei were counterstained with DAPI (250 ng/ml). Immunofluorescence staining was observed with an Axio fluorescence microscope (Zeiss, Jena, Germany). FITC-gelatin degraded area was measured by ImageJ software (National Institutes of Health, Bethesda, MD, USA) and normalized to DAPI intensity for quantitation of gelatin degradation.

### Preparation of conditioned medium and gelatin zymography

Subconfluent cells were incubated in serum-free medium for 24 h. The conditioned medium was collected by centrifugation at 2000 *g* for 5 min and secreted proteins in the supernatant were precipitated with cold trichloroacetic acid (TCA; Sigma-Aldrich). The precipitated proteins were analyzed using gelatin zymography as described previously [45].

### RNA isolation and reverse transcription (RT)-PCR analysis

Total RNA was isolated from each cell line using TRIzol reagent (Invitrogen). First-strand cDNA was synthesized from total RNA using oligo (dT)_15_ primers and the AMV RT system (Promega, Madison, WI, USA) according to the manufacturer's instructions. PCR was carried out under conditions of 27 cycles of denaturation at 94°C for 30 s, annealing (see Supplementary Table S3 for annealing temperatures) for 60 s, and extension at 72°C for 30 s. PCR products were analyzed by polyacrylamide gel electrophoresis (PAGE) through 5% gels. Real-time PCR was performed using the QuantiTect SYBR Green PCR Kit (Qiagen, Hilden, Germany) and the CFX Real-Time system (Bio-Rad, Hercules, CA, USA.).

### Dual-luciferase reporter assay

Cells (8 × 10^3^/well) were seeded in 96-well plates 16 h prior to transfection. Luciferase reporter construct pGL3-M9P-wt containing the human wild-type *MMP9* promoter or the promoterless vector pGL3-basic was cotransfected with reporter vector pRL-TK encoding *Renilla* luciferase using Lipofectamine 2000 [46]. After 24 h, luciferase activity was measured using a dual-luciferase reporter assay system (Promega) and normalized to *Renilla* luciferase activity.

### Fractionation of cytosolic and nuclear proteins

Subconfluent cells were separated into cytoplasmic and nuclear fractions as described previously [24], and proteins were analyzed by western blotting.

### Preparation of cell lysates, immunoprecipitation, and western blotting

Subconfluent cells were lysed with RIPA lysis buffer (50 mM Tris-HCl, pH 7.4, 150 mM NaCl, 1% NP-40, 0.5% sodium deoxycholate, and 0.1% SDS) containing 1 mM NaF, 1 mM Na_3_VO_4_, and Sigmafast protease inhibitor tablets (2 mM AEBSF, 300 nM aprotinin, 130 μM bestatin, 1 mM EDTA, 14 μM E-64, and 1 μM leupeptin; Sigma-Aldrich). Immunoprecipitation and western blotting were performed as described previously [17].

### Gelatinolytic activity in 3D culture

3D cell culture in Matrigel (Phenol Red-Free; Corning Inc., Corning, NY, USA) containing fluorogenic DQ Gelatin was performed as previously described [47, 48]. Briefly, cells (5 × 10^4^/100 μl) were incubated in phenol red-free DMEM containing signaling inhibitors for 30 min, mixed with 100 μl of 6 mg/ml Matrigel containing 50 μg/ml DQ Gelatin and signaling inhibitors, and seeded in 13-mm glass-bottomed dishes (SPL Life Sciences, Pocheon-si, Gyeonggi-do, Korea) coated with 50 μl Matrigel. After polymerization of Matrigel, 2 ml culture medium containing signaling inhibitors were added, and the dishes were incubated at 37°C for 72 h. Cells were incubated with Hoechst 33258 (2 μg/ml) for 30 min to counterstain nuclei. Intensity of fluorescence generated by DQ Gelatin cleavage was determined and normalized to that of Hoechst 33258 staining using a confocal microscope (LSM700; Zeiss) and ImageJ software.

### Immunohistochemical (IHC) staining of ESCC tissue and statistical analysis

Preparation and IHC staining of ESCC tissue were performed as described previously [8]. Micrographs were taken of stained tissue and evaluated for staining intensity by a pathologist blinded to the identity of the samples. Intensity of PTK7 or MMP-9 staining was scored on a scale of 0 (low) to 4 (high).

Statistical analysis of association of PTK7 and MMP-9 expression was carried out using Spearman's rank correlation. Correlation and statistical comparisons were performed using the R package ‘pspearman’ (version 0.3–0).

### Statistical analysis

All data subjected to statistical analysis were obtained from at least three independent experiments and are expressed as mean ± standard deviation. Statistical significance was analyzed by Student's *t-test* unless specified otherwise. A *p-value* < 0.05 was considered significant.

## SUPPLEMENTARY MATERIALS TABLES AND FIGURE


