# Supplementary material for: Catalytically defective receptor protein tyrosine kinase PTK7 enhances invasive phenotype by inducing MMP-9 through activation of AP-1 and NF-κB in esophageal squamous cell carcinoma cells
Source: Oncotarget. 2016 Sep 28;7(45):73242–56. doi: 10.18632/oncotarget.12303 (PMC5341976; doi:10.18632/oncotarget.12303)
Supplement: Supplementary file 1 [file oncotarget-07-73242-s001.pdf]

Catalytically defective receptor protein tyrosine kinase PTK7 enhances invasive phenotype by inducing MMP-9 through activation of AP-1 and NF-κB in esophageal squamous cell carcinoma cells

Supplementary Materials

Supplementary Table S1: Primer sequences for site-directed mutagenesis of mouse Src cDNA

Name	Nucleotide Sequence[†]	Nucleotide Position	GenBank Accession No.
**mSrc K297R**	F: 5′-GTTGCCATCAGAACTCTGAAGCCAGGCACC-3′<br>R: 5′-GACATGGTGCCTGGCTTCAGAGTTCTGATG-3′	1282–1311	NM_009271
**mSrc Y529F**	F: 5′-CTGAGCCACAGTTCCAGCCCGGGGAGAACC -3′<br>R: 5′-ATAGGTTCTCCCCGGGCTGGAACTGTGGCT-′3	1976–2005	NM_009271

[†]F, forward; R, reverse.

Supplementary Table S2: Oligonucleotide sequences for PTK7-knockout LentiCRISPRv2 vector

Name	Nucleotide Sequence[†]	Nucleotide Position*	GenBank Accession No.
**PTK7-sgRNA-F369**	F: 5′-CACCGCACGGAGCGGCGTTTCGCCC-3′<br>R: 5′-AAACGGGCGAAACGCCGCTCCGTGC-3′	369–388	U40271
**PTK7-sgRNA-F849**	F: 5′-CACCGGGTAGTAGCGAGGTATGAGG-3′<br>R: 5′-AAACCCTCATACCTCGCTACTACCC-′3	849–868	U40271

[†]F, forward; R, reverse. gRNA target sequence is underlined.
*Nucleotide position of gRNA target sequence is shown.

Supplementary Table S3: Primer sequences for RT-PCR analysis of *MMP2*, *MMP9*, *PTK7*, and *GAPDH* mRNA

Gene	Nucleotide Sequence[†]	Nucleotide Position	Annealing Temp. (°C)	GenBank Accession No.
MMP2	F: 5′-AGATCTTCTTCTTCAAGGACCGGTT-3′<br>R: 5′-GGCTGGTCAGTGGCTTGGGGTA-3′	1755–1779<br>1979–1958	62	NM_004530
MMP9	F: 5′-TGGGCTACGTGACCTATGAC-3′<br>R: 5′-CAAAGGTGAGAAGAGAGGGC-3′	2100–2119<br>2290–2271	59	NM_004994
PTK7	F: 5′-AGAGATGCCCCATGGTGGGC-3′<br>R: 5′-ACGGCTTGCTGTCCACGGTG-3′	3156–3176<br>3359–3339	55	U40271
GAPDH	F: 5′-ACTGCTTAGCACCCCTGGCCA-3′<br>R: 5′-TTGGCAGTGGGGACACGGAAG-3′	488–508<br>740–720	62	BC023632

[†]F, forward; R, reverse.

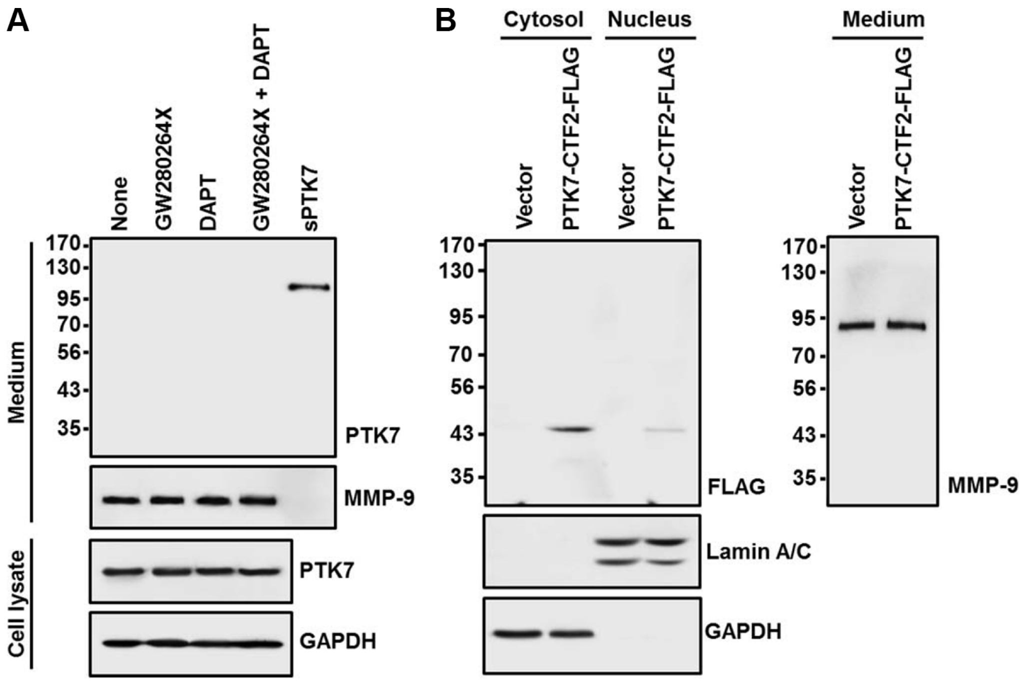

Supplementary Figure S1: MMP-9 expression in PTK7 shedding inhibitor-treated or PTK7-CTF2-expressed TE-10 cells. (**A**) Subconfluent TE-10 cells were incubated in serum-free medium for 24 h in the presence of GW280264X (selective ADAM10 and ADAM17 inhibitor, 1 μM) and/or DAPT (γ-secretase inhibitor, 10 μM). (**B**) TE-10 cells were transfected with pcDNA3.1 (Vector) or pcDNA3.1-PTK7-CTF2-FLAG (PTK7-CTF2-FLAG). Cells were incubated with serum-free media for 24 h and were lysed with RIPA lysis buffer (A) or separated into cytoplasmic and nuclear fractions (B). Conditioned media were concentrated by TCA precipitation. Proteins in the concentrated media, cell lysates or cell fractions were analyzed by western blotting using indicated antibodies. sPTK7 (25 ng) was used as a control.
